# Microbiome Structure of a Wild *Drosophila* Community along Tropical Elevational Gradients and Comparison to Laboratory Lines

**DOI:** 10.1128/aem.00099-23

**Published:** 2023-05-08

**Authors:** Joel J. Brown, Anna Jandová, Christopher T. Jeffs, Megan Higgie, Eva Nováková, Owen T. Lewis, Jan Hrček

**Affiliations:** a University of South Bohemia, Faculty of Science, České Budějovice, Czech Republic; b Institute of Entomology, Biology Centre of the Czech Academy of Sciences, České Budějovice, Czech Republic; c Department of Zoology, University of Oxford, Oxford, United Kingdom; d College of Science & Engineering, James Cook University, Townsville, Queensland, Australia; e Institute of Parasitology, Biology Centre of the Czech Academy of Sciences, České Budějovice, Czech Republic; University of Michigan-Ann Arbor

**Keywords:** bacteria, community, *Drosophila*, ecology, elevation gradient, field and laboratory, metabarcoding, microbiome, symbiosis

## Abstract

Variation along environmental gradients in host-associated microbial communities is not well understood compared to free-living microbial communities. Because elevational gradients may serve as natural proxies for climate change, understanding patterns along these gradients can inform our understanding of the threats hosts and their symbiotic microbes face in a warming world. In this study, we analyzed bacterial microbiomes from pupae and adults of four *Drosophila* species native to Australian tropical rainforests. We sampled wild individuals at high and low elevations along two mountain gradients to determine natural diversity patterns. Further, we sampled laboratory-reared individuals from isofemale lines established from the same localities to see if any natural patterns are retained in the lab. In both environments, we controlled for diet to help elucidate other deterministic patterns of microbiome composition. We found small but significant differences in *Drosophila* bacterial community composition across elevation, with some notable taxonomic differences between different *Drosophila* species and sites. Further, we found that field-collected fly pupae had significantly richer microbiomes than laboratory-reared pupae. We also found similar microbiome composition in both types of provided diet, suggesting that the significant differences found among *Drosophila* microbiomes are the products of surrounding environments with different bacterial species pools, possibly bound to elevational differences in temperature. Our results suggest that comparative studies between lab and field specimens help reveal the true variability in microbiome communities that can exist within a single species.

**IMPORTANCE** Bacteria form microbial communities inside most higher-level organisms, but we know little about how the microbiome varies along environmental gradients and between natural host populations and laboratory colonies. To explore such effects on insect-associated microbiomes, we studied the gut microbiome in four *Drosophila* species over two mountain gradients in tropical Australia. We also compared these data to individuals kept in the laboratory to understand how different settings changed microbiome communities. We found that field-sampled individuals had significantly higher microbiome diversity than those from the lab. In wild *Drosophila* populations, elevation explains a small but significant amount of the variation in their microbial communities. Our study highlights the importance of environmental bacterial sources for *Drosophila* microbiome composition across elevational gradients and shows how comparative studies help reveal the true flexibility in microbiome communities that can exist within a species.

## INTRODUCTION

Patterns of diversity over environmental gradients like latitude, elevation, or environmental degradation have long been of interest in community ecology and are of renewed interest for studying the potential consequences of climate change (1–5). Most studies have focused on animals and plants to investigate these patterns, but bacterial communities are receiving increased attention. Some studies suggest free-living bacteria do not follow the same broad biogeographic patterns as plants and animals (6–8). Fierer et al. (1) showed that soil bacteria did not change significantly in diversity when sampled across an elevational gradient, in contrast to trends documented in most other taxa. Subsequent studies have found inconsistent patterns in bacterial communities sampled from streams and soils across elevational gradients, with differences usually being attributed to changes in pH and C/N ratio (2, 8–10).

Many insects maintain intimate communities of symbiotic microbes (their “microbiome”). Insect microbiomes can play important roles in host health, digestion, thermal regulation, and protection against natural enemies (reviewed in references 11 to 13). In turn, many factors can influence insect microbiome composition, some host dependent (e.g., diet, insect species identity, ontogeny, and parent-to-offspring transmission) and others host independent (e.g., abiotic factors like local environment and temperature) (14–22). Symbioses between insects and bacteria have been particularly well investigated (23), notably because insect microbiome communities tend to be less complex than those of vertebrates (24). However, in contrast to environmental microbial communities, the effect of elevational change on insect-associated microbiomes has yet to be investigated in depth. The most conspicuous aspect of a change in elevation is a difference in mean temperature, creating different environments that can be used as a proxy for climate change scenarios (25, 26). Elevational differences in temperature mean we would expect to see differences in microbiome composition related to temperature-dependent development in both insects (27–31) and bacteria (32–34). Thus, at different elevations and in climate change scenarios, insect-associated microbiomes could have different compositions (20) with potentially important consequences on microbiome and host functionality.

*Drosophila* spp. are established models for studying insect-associated microbiomes (35–40) because they are cosmopolitan, occur in a wide variety of habitats, and are easy to maintain in laboratory cultures. *Drosophila*-associated microbiomes have important functional impacts on many aspects of their ecology, including thermal tolerance (41), development (42), ability to recognize kin (43), and immunity (38, 44). The microbiomes are of moderate-to-low diversity, making them relatively simple to characterize. Additionally, some *Drosophila* species and populations possess intracellular bacterial symbionts (*Wolbachia* and *Spiroplasma*) that can influence host immunity and protect against natural enemies, including pathogenic fungi, nematodes, and parasitoids (45–49). This, combined with the well-studied nature of *Drosophila*, makes them ideal candidates for investigating insect-associated microbiomes over elevational gradients.

Here, we examine the effects of elevation change on insect microbiome composition by focusing on the underlying biotic and abiotic factors, including elevation, host species identity, and site location. We sampled wild populations of four focal species of frugivorous *Drosophila* from two mountain gradients in Queensland, Australia, Drosophila rubida, D. pseudoananassae, D. pallidifrons, and D. sulfurigaster. These species occur throughout north Queensland along multiple altitudinal gradients in the wet tropics. We chose these four species because they are abundant and occur in sympatry across the full elevational gradient at our focal sites (50). We hypothesized that variation in microbiome composition between high- and low-elevation populations will reflect temperature differences at these sites. To control for diet in the field, we exclusively sampled pupae from banana-baited bottle traps (see Jeffs et al. [50]). The sampling approach guarantees that each analyzed individual originated from an egg laid in our bottle traps and therefore fed solely on yeasted banana. To reinforce our investigation, we analyzed lab-reared flies of the same species collected from the same field sites to test if their microbiomes retained any natural differences when reared in the laboratory on a standard yeast-based diet. We expected *a priori* to find high among-individual variation and hypothesized that species identity, elevation, and environment of origin (i.e., lab versus field) would be the primary causes of the difference in host microbiome composition (35, 39, 40, 51).

## RESULTS

**FIG 1:**
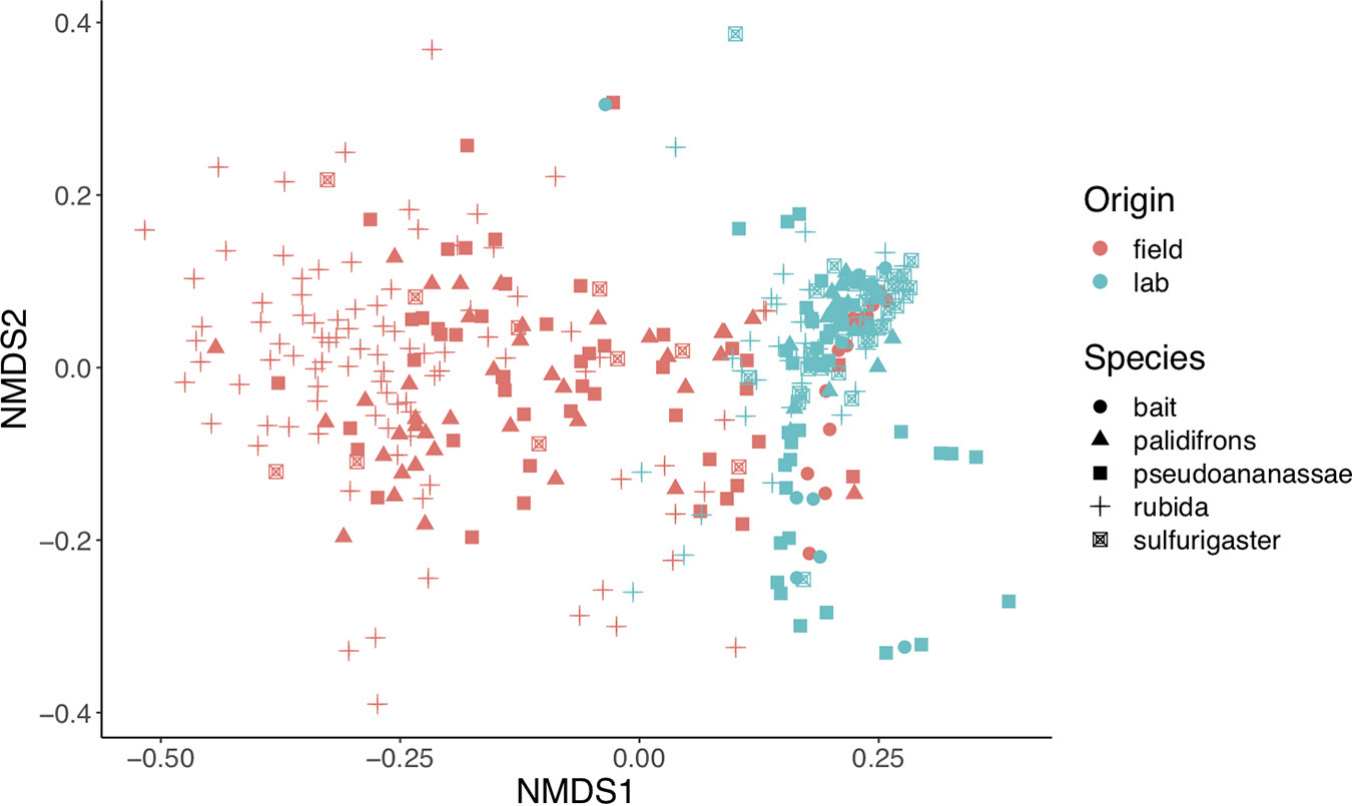
NMDS analysis of microbiome communities from all samples in this study, from the lab (blue) and the field (red). Samples of Drosophila rubida, D. pseudoananassae, D. pallidifrons, D. sulfurigaster, and food bait are indicated by different shapes.

**FIG 2:**
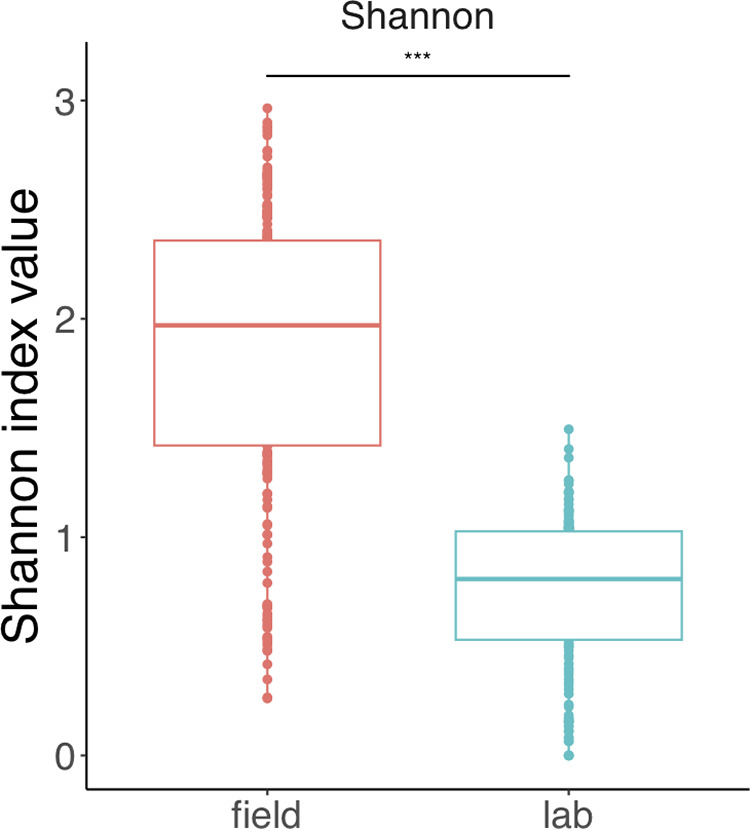
Comparison of Shannon index values for all samples in this study, split by environment of origin. Lab samples are shown in blue and field samples in red. The triple asterisk shows high significance (*P* > 0.001) of a paired Wilcoxon test.

We first tested which of the studied factors influenced microbiome composition in all core field and lab samples combined, using permutational multivariate analysis of variance (PERMANOVA) on Bray-Curtis values. The dominant explanatory variable was environment of origin (i.e., whether a sample came from the lab or field; nonmetric multidimensional scaling ordinations [NMDS] mean stress ≈ 0.15; PERMANOVA *R*^2^ = 0.150; Benjamini-Hochberg corrected *P* ≤ 0.001; beta-dispersion *F* = 126.8, *P* ≤ 0.001) (Fig. 1 and see Table S1 in the supplemental material). The *R*^2^ values (see Table S1) indicated that environment of origin was clearly an overarching explanatory variable; thus, we decided to further analyze the field and lab samples separately to establish the important deterministic factors within each environment. The main trend in our results was a significant reduction in microbiome richness in lab-reared flies of all species based on a paired Wilcoxon rank sum test between Shannon index values for lab and field samples (Benjamini-Hochberg corrected *P* ≤ 0.001) (Fig. 2). This significant trend held when pupae or adult *Drosophila* were analyzed separately (Fig. S3 to S5).

### Microbiome patterns from field samples.

**FIG 3:**
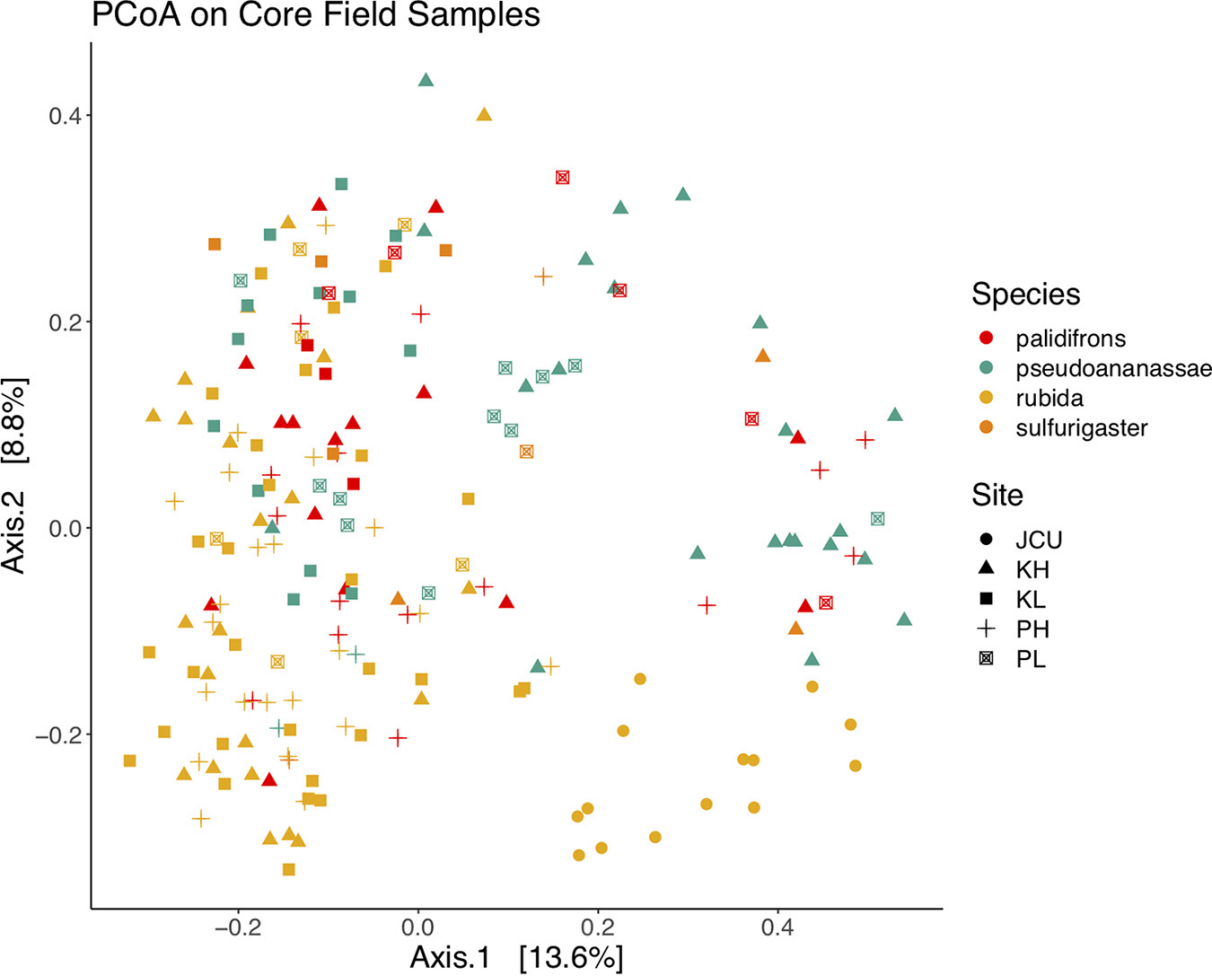
PCoA analysis of microbiome communities from the core field samples. Samples of Drosophila rubida, D. pseudoananassae, D. pallidifrons, and D. sulfurigaster are indicated by different colors, and the different sites of collection are indicated by shape. K, Kirrama; P, Paluma; JCU, James Cook University; L, low (elevation); H, high (elevation). Samples of D. rubida collected at JCU were all adults.

**FIG 4:**
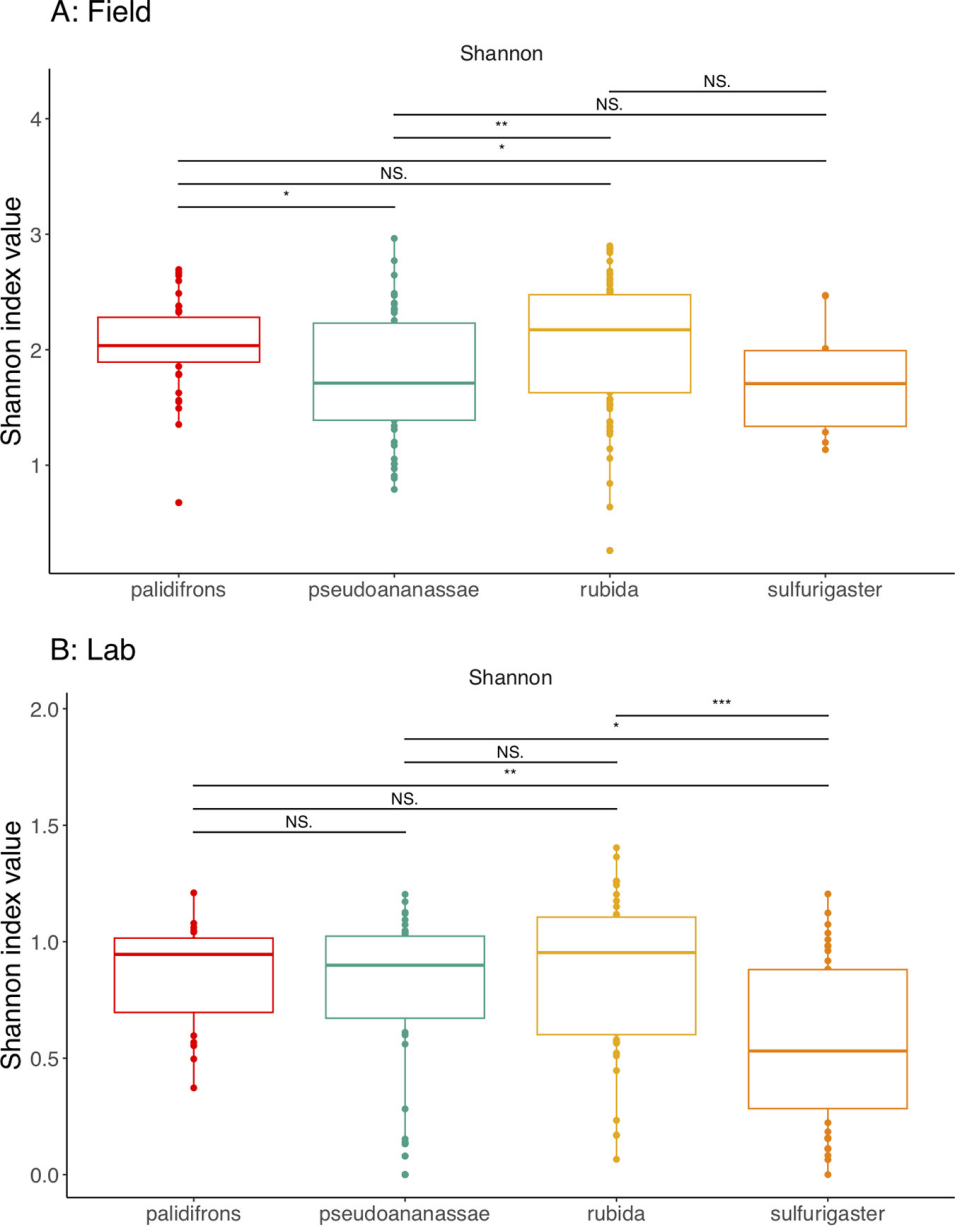
Comparison of Shannon index values from each species of *Drosophila* from the field (A) and lab environments (B). Each color represents a different species. Statistical comparisons come from paired Wilcoxon tests. Three asterisks denote a highly significant result (*P* ≤ 0.001). Two asterisks indicate a result of moderate significance (between *P* < 0.1 and *P* ≤ 0.001). One asterisk denotes a marginally significant result (*P* < 0.1). NS, not significant.

**FIG 5:**
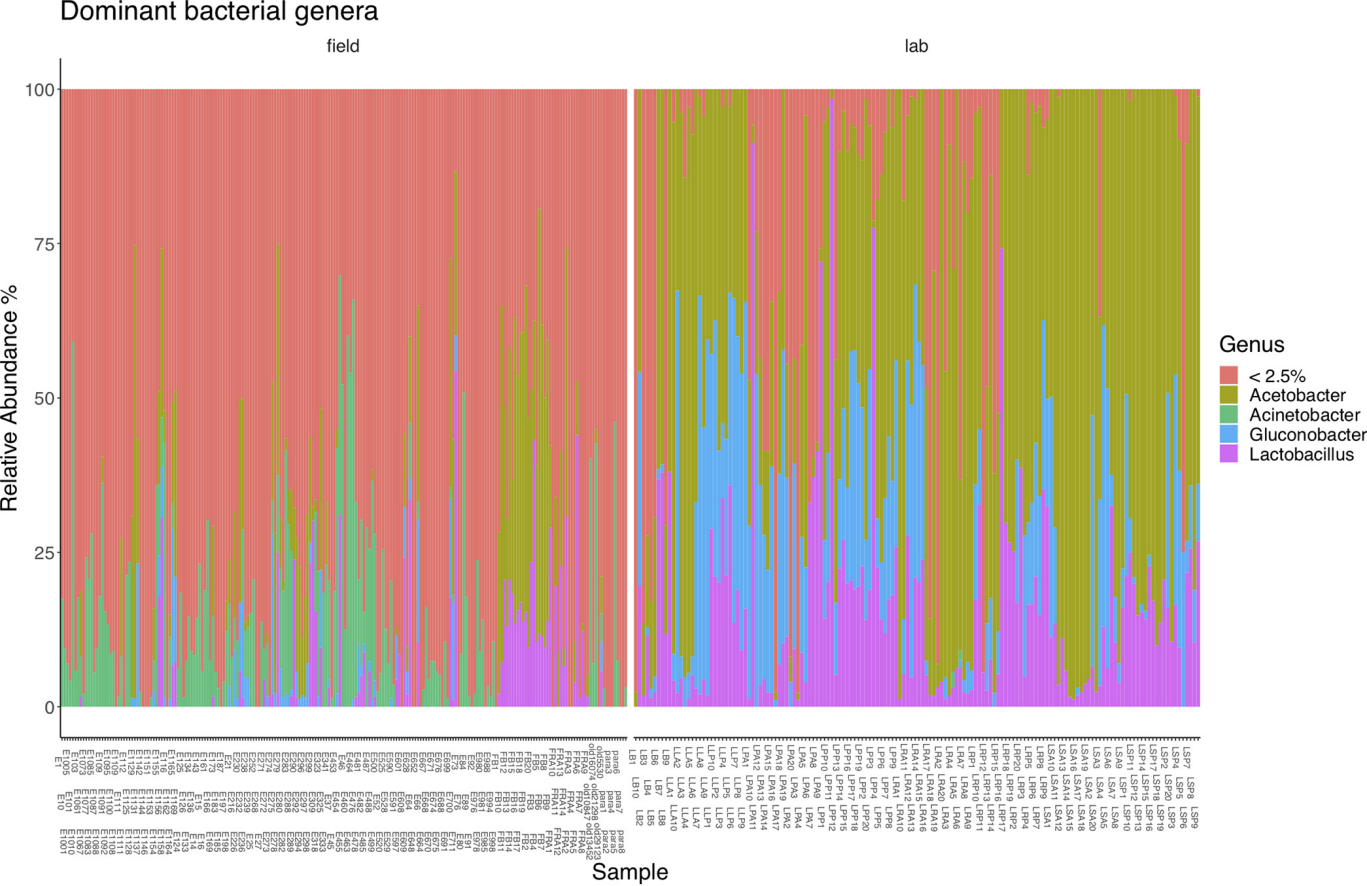
The most abundant bacterial genera from all samples in this study. Different colors mark different bacterial genera. Less than 2.5% is a conglomerate category of low abundance taxa that made up less than 2.5% of the median number of reads. Each individual column represents an individual sample. Relative abundance is on the *y* axis.

When focusing on core field samples, the main factors explaining variation in microbiome structure were trap identity (PERMANOVA *R*^2^ = 0.13, Benjamini-Hochberg corrected *P* ≤ 0.001, beta-dispersion *P* ≤ 0.001) and the interaction between site and trap (PERMANOVA *R*^2^ = 0.13, Benjamini-Hochberg corrected *P* ≤ 0.001, beta-dispersion *P* ≤ 0.001) (Table S2), suggesting that local environmental differences in site location, as well as trap identity, have a significant effect on *Drosophila* microbiome composition (Fig. 3). Elevation alone explained a small, but still significant, proportion of the variation observed (*R*^2^ = 0.03). The banana-baited bottle traps were left exposed in the field for different durations (between 11 and 24 days) to ensure we characterized the *Drosophila* community fully. PERMANOVA results suggest that the length of exposure had a significant influence on microbiome composition but was of lower importance than the other factors we identified based on the amount of variation explained (*R*^2^ = 0.03). Based on the PERMANOVA analysis, field site was a significant variable but only explained 7% of the variation in diversity (reflected in the minimal differences in average Shannon index value in Fig. S6). When comparing pupae samples and bait samples, the main explanatory variable was sample type, i.e., whether a sample came from *Drosophila* or a piece of banana bait (PERMANOVA *R*^2^ = 0.16, Benjamini-Hochberg corrected *P* ≤ 0.001, beta-dispersion *P* ≤ 0.001) (Fig. S7). We also found evidence of *Drosophila* species-specific differences in microbiome diversity, based on paired Wilcoxon tests between Shannon index values for different species (*P* ≤ 0.001) (Fig. 4). DeSeq analysis of differential abundance indicated some bacterial genera were significantly more abundant in some *Drosophila* species but not others, most prominently *Kozakia* and *Corynebacterium* (Table S3). The most abundant bacterial genera (Fig. 5) were evenly distributed throughout all four *Drosophila* species sampled here, including Acinetobacter, which was the most dominant genus overall.

### Microbiome patterns from laboratory samples.

**FIG 6:**
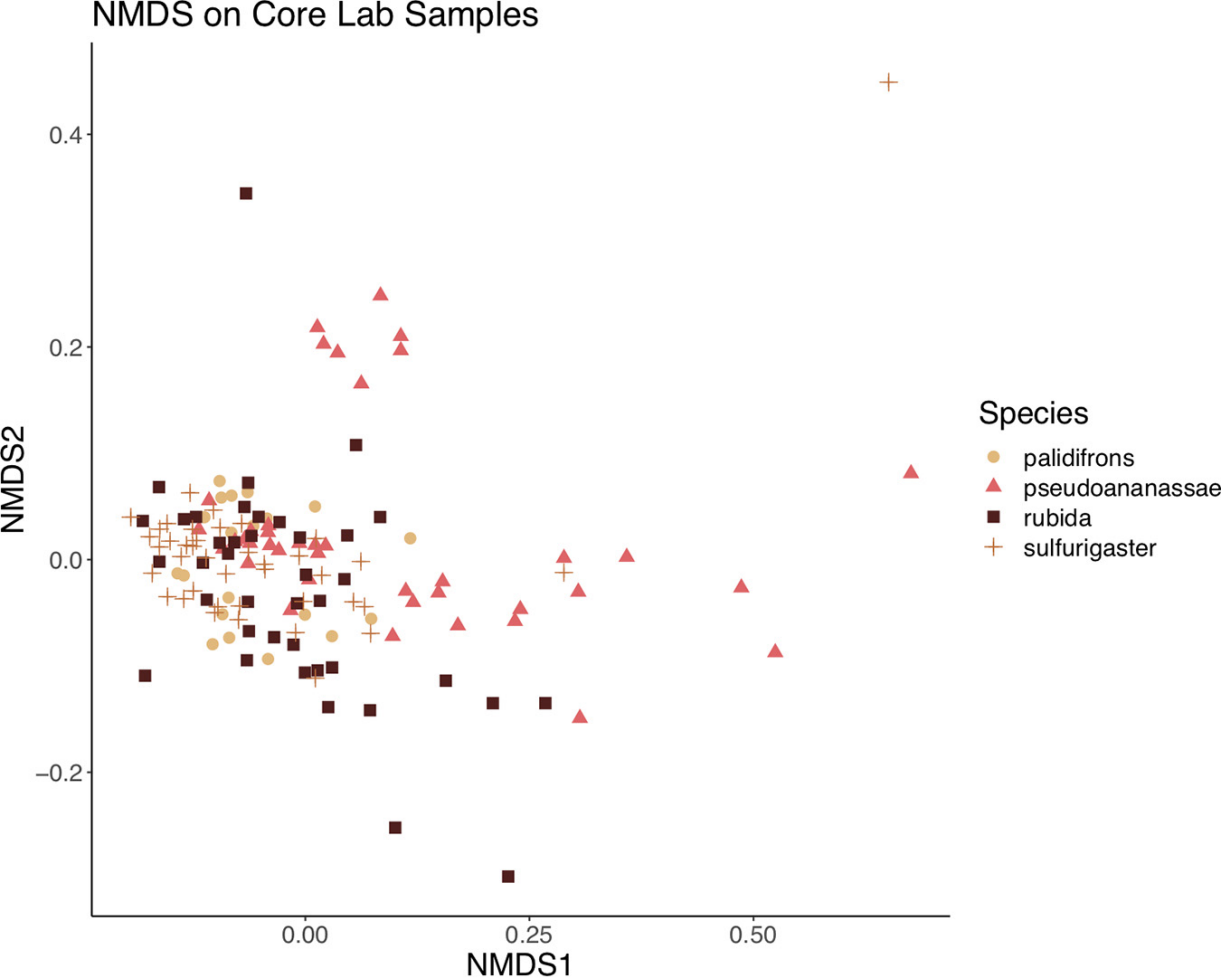
NMDS analysis of microbiome communities from core lab samples. Samples of Drosophila rubida, D. pseudoananassae, D. pallidifrons, and D. sulfurigaster are indicated by different colors and shapes.

The main factors explaining variation in microbiome structure from the lab-reared samples were *Drosophila* species identity (PERMANOVA *R*^2^ = 0.11, Benjamini-Hochberg corrected *P* ≤ 0.001, beta-dispersion *P* ≤ 0.001), isofemale (IF) line (PERMANOVA *R*^2^ = 0.13, Benjamini-Hochberg corrected *P* ≤ 0.001, beta-dispersion *P* ≤ 0.001), and the number of generations an isofemale line had been in the lab (PERMANOVA *R*^2^ = 0.12, Benjamini-Hochberg corrected *P* ≤ 0.001, beta-dispersion *P* = 0.007) (Table S4), suggesting that the duration a lineage of flies had been in the lab environment was important for explaining microbiome composition. While the alpha diversity differences between species were not significant (Shannon index values) (Fig. 6), the amount of variation explained by species-specific differences in the PERMANOVA analysis indicates that microbiome composition across species differed. All four *Drosophila* species contained high proportions of *Acetobacter*, *Lactobacillus*, and *Gluconobacter*, but only D. pseudoananassae contained *Wolbachia* (Fig. S8). The interaction between isofemale line and generation was also highly significant and explained a high proportion of variation relative to other factors, which was expected because IF lines were established at different times and therefore had been in the lab for various numbers of generations at the time of sampling. Life stage (i.e., the difference between pupae and adults) was not one of the most important explanatory factors based on the amount of variation explained (PERMANOVA *R*^2^ = 0.06). There was no detectable effect of elevation (PERMANOVA *R*^2^ = 0.01), i.e., the elevation of the field site where an isofemale line was collected did not have a significant effect on the microbiome structure in the lab.

### Comparisons between field and laboratory samples and microbiome of the food source.

There was no significant difference in microbiome alpha diversity of laboratory food samples from the Czech Republic and the banana bait that we used in the field in Australia (paired Wilcoxon test on Shannon index values, *P* = 0.09) (Fig. S9). Banana bait and lab fly food both contained high relative proportions of *Acetobacter* and *Lactobacillus*, with some lab food samples containing *Gluconobacter* (Fig. S10). Despite the physical differences in food sources, the most abundant bacterial genera were the same, and the food source microbiome diversity was low. In lab-reared flies, these three bacterial genera dominated the microbiomes of pupae and adults (Fig. S7). In the field, however, *Acetobacter* and *Lactobacillus* were not the most abundant genera of either pupae or adult gut microbiomes. *Acetobacter* and *Lactobacillus* had the greatest difference in relative abundance between field and lab samples (summary statistics in Table 1 and Fig. S11). While still present in field samples, the relative abundance of *Acetobacter* and *Lactobacillus* was proportionally lower in their more taxon-rich microbiomes (Fig. 5).

**TABLE 1:** Summary output of DeSeq differential abundance analysis showing the top 5 bacterial genera by origin of sample^*a*^

Genus	Base mean	Log_2_ fold change	lfcSE^*b*^	Test statistic	Adjusted *P* value
*Acetobacter*	436.99	3.59	0.38	9.46	≤0.001
*Lactobacillus*	183.75	3.16	0.38	8.34	≤0.001
Acinetobacter	195.62	−7.91	0.33	−23.70	≤0.001
Klebsiella	105.22	−7.01	0.37	−18.99	≤0.001
*Chishuiella*	59.69	−6.19	0.41	−14.97	≤0.001

a Positive test statistic values indicate a genus significantly more abundant in lab samples, and negative test statistics indicate a genus significantly more abundant in field samples.

b lfcSE, standard error of the log_2_ fold change.

### Robustness of results.

There was no detectable difference in microbiome alpha diversity of parasitized pupae of D. rubida compared to unparasitized pupae (paired Wilcoxon test on Shannon index values, *P* = 0.25) (Fig. S12), nor were there bacterial genera unique to parasitized samples. We also found that the 99% identity operational taxonomic unit (OTU) table produced qualitatively the same results as the 97% identity OTU table. For example, in an NMDS on all samples, the dominant explanatory variable was still environment of origin (NMDS mean stress ≈ 0.17, PERMANOVA *R*^2^ = 0.17, Benjamini-Hochberg corrected *P* ≤ 0.001, beta-dispersion *P* ≤ 0.001) (Fig. S1). Removing *Wolbachia* from the data set also did not qualitatively change the outcome of statistical tests, e.g., the main factors explaining microbiome structure in lab-reared samples were *Drosophila* species identity (PERMANOVA *R*^2^ = 0.11, Benjamini-Hochberg corrected *P* ≤ 0.001, beta-dispersion *P* ≤ 0.001), isofemale line (PERMANOVA *R*^2^ = 0.17, Benjamini-Hochberg corrected *P* ≤ 0.001, beta-dispersion *P* ≤ 0.001), and the number of generations an isofemale line had been in the lab (PERMANOVA *R*^2^ = 0.12, Benjamini-Hochberg corrected *P* ≤ 0.001, beta-dispersion *P* ≤ 0.001).

## DISCUSSION

Our results revealed a small but significant variation in microbiome structure between *Drosophila* populations from high and low elevations across both gradients. We expected to find greater differences than we observed across elevation because there is well-documented evidence of both insects and bacteria developing differently according to differences in temperature of >5°C (29–31, 33, 34, 52). This finding could be a result of the *Drosophila* species sampled here being ubiquitous across elevations without forming sufficiently distinct populations at high and low elevation sites or because the ~5°C temperature shift between our sites was not strong enough to drastically alter microbiome composition.

The most pronounced difference in microbiome diversity was between individuals raised in the laboratory and those raised in the field. Interestingly, the two food sources (banana bait in the field and yeast-based *Drosophila* medium in the lab) had very similar microbiome profiles, suggesting that dietary factors were not primarily responsible for the observed differences in alpha diversity between environments. The bacterial community from lab food matches well with the microbiomes found within lab-reared pupae and adult flies. This was expected because it reflects a well-established pathway of insect microbiome colonization: *Drosophila* ingests food and acquire bacteria associated with that food source (36). Yet in the field, *Drosophila* microbiome diversity does not correspond well with the bacterial communities found on banana bait samples (see Fig. S7 in the supplemental material). The observed pattern can be explained by significant differences in microbiome colonization from environmental bacterial species pools (53, 54). The flies sampled from the lab come from a highly regulated environment, with a specific and consistent food source provided into heat-sterilized glass vials, so the only “available” bacteria for colonizing their microbiomes come from the diet, surrounding lab environment, and vertically inherited endosymbionts (e.g., *Wolbachia* in D. pseudoananassae). In contrast, the bacterial species pool in the Australian tropical rainforest comprises much greater diversity and abundance of different taxa, creating a greater variety of possible microbiome composition within *Drosophila* hosts. This diversity of taxa creates more room for ecological drift, dispersal, and selection to act on microbiome communities, in turn creating greater among-individual and between-species variation in wild flies. Consistently higher diversity in wild *Drosophila* microbiomes suggests that microbiomes are predominantly colonized from the wider environment and dependent on local species pool diversity. For instance, bottle traps were visited by other organisms, e.g., staphylinid beetles, neriid flies, and Lepidoptera, which could also have been a source of bacteria colonizing the microbiome of the *Drosophila* sampled in this study.

There was consistently low microbiome alpha diversity found in both lab-reared pupae and lab-reared adults, suggesting that low alpha diversity within pupae is an accurate representation of lab-reared microbiomes. This result was surprising because we anticipated some stage-specific microbiome community patterns given that *Drosophila* is holometabolous and thus undergoes substantial gut remodeling during complete metamorphosis (55). The consistency across life stages from lab-reared individuals provides further evidence for the depauperate nature of the lab microbial environment. In contrast to the lab, the field-caught adults of D. rubida lacked congruence with the field-caught pupae, and both life stages lack similarity with banana bait samples (Fig. S7 and S13). The lack of geographic variation in microbiome composition suggests that different sites are unlikely to fully explain the discrepancy. With adult flies, we cannot rule out that they might have fed on a substance other than our yeasted banana bait prior to arriving at our bottle traps, making dietary variation a parsimonious explanatory factor in life stage differences (36, 56). It is also possible that the high alpha diversity we found in pupal samples reflects their metabolic activity (despite their lack of feeding), given that complete metamorphosis is an intense period of organismal change (57). The substantial differences in microbiomes between lab and field specimens suggest that future studies should be cautious and specific with the types of microbiome-related questions investigating laboratory flies. Interpreting microbiome community composition from lab-kept specimens, particularly those from cultures maintained across multiple generations, is unlikely to yield data entirely representative of natural microbiomes (58, 59), except perhaps in scenarios where microbiome communities from lab-reared specimens are a subset of the more diverse microbiomes found in the field (NMDS mean stress ≈ 0.15, PERMANOVA, *R*^2^ = 0.150, Benjamini-Hochberg corrected *P* ≤ 0.001, beta-dispersion *F* = 126.8 and *P* ≤ 0.001) (Table S1 and Fig. 1).

Previous studies on *Drosophila* have demonstrated high intra- and interspecific variation in microbiome community composition from both wild-caught and lab-reared flies (39, 40, 58, 60, 61), which our results corroborate. Controlling for diet in both scenarios allowed us to recognize this species specificity more accurately. We found a significant effect of species identity (11% variation explained), but it did not explain as many variations as the results from Adair et al. (40), who found species identity explaining 42% and 70% variation in two different sets of *Drosophila* spp. This discrepancy could be a product of the species themselves (i.e., we used a different set of *Drosophila* species) or the number of species studied (we studied four species here; Adair et al. [40] studied 18), but the evidence from both studies suggests that species specificity is maintained in the lab, albeit not in a consistent manner. Statistically significant differences in microbiome alpha diversity measures between different *Drosophila* species in the field were not reflected in the lab. From the field, the only significant difference in microbiome richness was between D. pseudoananassae and D. rubida, and this was nonsignificant in the lab. Moreover, there was a highly significant difference in richness between D. rubida and D. sulfurigaster in the lab, but not in the field. Some of our significant PERMANOVA results were accompanied by significant beta-dispersion, which suggests that there was some heterogeneous variation in the variables we measured. This was expected based on the high variation typically exhibited by microbiome community data and typically found when sampling wild individuals. Even with a greater and more even sample size, we would still expect to find a large, significant difference in community richness and composition between field and lab *Drosophila* microbiomes due to the general depletion of microbial alpha diversity in lab-reared isofemale lines.

In other insect species, host transmission of extracellular symbionts (like those in the gut) has been hypothesized to result in long-term associations between insect and microbe (62–64). The long-term laboratory survival of our four *Drosophila* species (minimum of 24 generations) with radically different microbiome composition (compared to their counterparts from the field) suggests that their symbiotic relationship with bacteria is not highly specialized (65), corroborating previously published findings. For example, Wong et al. (37) found no consistent evidence for a core microbiome in multiple *Drosophila* species, and Storelli et al. (66) described axenic *Drosophila* returning to normal growth and development in the presence of a single bacteria species. Furthermore, Coon et al. (67, 68) showed that axenic mosquito larvae do not develop past the first instar, and colonization by different living bacteria can facilitate successful development. Thus, our results provide further evidence of satisfactory microbiome function being provided by a limited number of bacterial species, which poses questions about the advantages of diverse microbiomes within wild insects and the links between microbiome community diversity and host fitness.

Overall, we found significant differences in microbiome diversity of field-caught and lab-reared *Drosophila*, which were consistent across species and life stages. We hypothesize that these differences in diversity are the products of environments with markedly different bacterial species pools. To elucidate functional conclusions from insect-microbiome analyses, more in-depth molecular analysis (e.g., metagenomics, transcriptomics) is required. We suggest that comparative studies between lab and field specimens help reveal the true variability in microbiome communities that can exist within a single species.

## MATERIALS AND METHODS

### Study sites.

The Australian Wet Tropics World Heritage Area is a 450-km-long, narrow section of rainforest along Queensland’s northeast coast between Cooktown and Townsville (15 to 19°S, 145 to 146.30°E). Samples were collected from two altitudinal gradients, Paluma Range Road (within Paluma Range National Park; 19°00′S, 146°14′E) and Kirrama Range Road (within Girramay National Park; 18°12′S, 145°50′E). The Paluma gradient ranges from 59 m to 916 m above sea level (a.s.l.), and the Kirrama gradient ranges from 92 m to 770 m a.s.l (50). We chose sites at high and low elevations (Paluma, 880 m and 70 m; Kirrama, 730 m and 70 m, respectively) to capture an ~5°C temperature range (mean temperatures, 21°C at high elevation, 26°C at low elevation) (50). Temperature was recorded by multiple dataloggers suspended next to bottle traps at each site, which took a reading every hour. Our previous study on these gradients determined an ~5°C temperature range and found substantial differences in *Drosophila* community composition at high and low elevations, with some species not present at the low and high ends of the gradient and others changing in abundance (50).

### Sample collection and selection.

To ensure a comparable data set, we selected stratified subsets of samples from the field and laboratory. We primarily collected pupae because they are more easily standardized than larvae and allow us to account for discrepancies in development rates between species. We additionally collected a small number of adult flies to compare microbiome composition between pupae and adults. Based on the results of Jeffs et al. (50), which used pupae samples to identify the natural *Drosophila*-parasitoid food web with cytochrome oxidase I (COI) metabarcoding and multiplex PCR methods, we selected 214 field samples of the 4 most abundant *Drosophila* species that occurred at all elevations along both transects, Drosophila rubida, D. pseudoananassae, D. pallidifrons, and D. sulfurigaster (Table 2). Eight D. rubida pupae were parasitized by parasitoid wasps, enabling us to test if there are any changes in microbiome richness or unique microbial taxa associated with a developing parasitoid. We subsequently sampled 70 pupae and 70 adults from isofemale (IF) laboratory lines (2 to 4 lines per species) of these four elevationally ubiquitous species (20 pupae and 20 adults from D. sulfurigaster, D. rubida, and D. pseudoananassae and 10 pupae and 10 adults from D. pallidifrons) to investigate if suspected natural patterns (site- and species-specific influence) were retained in lab-reared flies. Additionally, we took 10 samples of the food source provided to lab-reared *Drosophila* and 20 samples of the banana bait we used in our field sampling to compare *Drosophila* microbiome samples to a dietary reference and determine how congruent the microbiome communities were between the food source and the insect hosts. Table 2 presents a detailed breakdown of all samples used in this study.

**TABLE 2:** Breakdown of the sample set used in this study^*a*^

Species	Stage	Environment of origin	Site(s)	No. of samples	Core or additional
D. rubida	Pupae	Field	PL, PH, KL, KH	71	Core
D. rubida (parasitized)	Pupae	Field	PL, PH, KL, KH	8	Core
D. rubida	Adult	Field	JCU	14	Core
D. pseudoananassae	Pupae	Field	PL, PH, KL, KH	48	Core
D. pallidifrons	Pupae	Field	PL, PH, KL, KH	39	Core
D. sulfurigaster	Pupae	Field	PL, PH, KL, KH	10	Core
D. rubida	Pupae	Lab	PL, PH, KL, KH	20	Core
D. rubida	Adult	Lab	PL, PH, KL, KH	20	Core
D. pseudoananassae	Pupae	Lab	PL, KL, KH	20	Core
D. pseudoananassae	Adult	Lab	PL, KL, KH	20	Core
D. pallidifrons	Pupae	Lab	PH, KH	10	Core
D. pallidifrons	Adult	Lab	PH, KH	10	Core
D. sulfurigaster	Pupae	Lab	PL, PH, KL, KH	20	Core
D. sulfurigaster	Adult	Lab	PL, PH, KL, KH	20	Core
Banana bait	NA	Field	PL, PH, JCU	20	Additional
Lab fly food	NA	Lab	NA	10	Additional

a PL, Paluma low; PH, Paluma high; KL, Kirrama low; KH; Kirrama high; JCU, James Cook University campus in Townsville; NA, not available.

The *Drosophila* pupae field samples were collected from banana-baited bottle traps placed at low- and high-elevation sites along both altitudinal gradients. Each bottle trap had a piece of cardboard to assist *Drosophila* larvae in pupation. Bottle traps were left exposed for either 11 to 12, 14 to 15, or 24 days to capture the natural variation in community colonization and ontogenetic development in different *Drosophila* species (50). On the day of sampling, these cards were removed and sealed in Ziploc bags. The individuals we collected as pupae thus only fed on banana bait. Pupae from each card were sampled by placing the card on a white plastic dinner plate and adding distilled water, using a small paintbrush to remove all pupae. Each pupa was placed into an individual well in 96-well PCR plates and preserved in 100% ethanol. Adults were aspirated from bottle traps hung at James Cook University, Townsville (JCU), 2 days after provision of fresh banana bait and placed into individual vials in 100% ethanol. JCU became a supplementary sampling site after Kirrama became inaccessible due to heavy rainfall and landslides. Laboratory-reared pupae were collected with forceps from standard fly food. Adults were collected with an aspirator and sexed and then placed in individual vials with 100% ethanol. Laboratory isofemale lines were established from the same populations sampled in the field (i.e., they were collected at the same sites and shipped live to the lab in the Czech Republic from 2017 to 2018, after collection of field samples in 2016). Isofemale lines were kept in the lab on a standard *Drosophila* diet medium (corn flour, sugar, agar, yeast, and methyl-4-hydroxybenzoate; see complete recipe in the supplemental information) for between 18 and 30 months by the time of sampling.

Sample DNA was individually extracted using single-column GeneAid blood and tissue kits according to the manufacturer’s instructions, with 1 extraction negative control accompanying every 29 samples. For confirmation of *Drosophila* species identification, we utilized a multiplex PCR approach (50). The PCRs were based on the internal transcribed spacer 2 (ITS2) and COI regions using custom specific forward primers and a universal reverse primer. Each species of *Drosophila* gives a different length of product; thus, we combined species-specific primers into 4 different multiplex PCRs. Gel electrophoresis of PCR products showed differences in product length, allowing us to identify species. Multiplex PCR was inconclusive between two closely related species, Drosophila sulfurigaster and D. pallidifrons, and D. bipectinata and D. pseudoananassae, and we therefore used Sanger sequencing with custom Diptera-specific primers based on the ITS2 region (ITS2DipF106 [TGCTTGGACTACATATGG] with two reverse primers, ITS2DipR240-1 [ATTTTTTATGCTAGACATTTCTC] and ITS2DipR240-2 [TTTTTATGCTAGACATTCCTC], giving a final product of 280 bp) to identify these samples to species. Full details of these processes and primer sequences can be found in the supporting information of Jeffs et al. (50).

### Library preparation and sequencing.

After extraction and identification, all samples were moved to 96-well plates in a randomized order in preparation for bacterial sequencing. DNA templates were stored at −75°C. These templates were used for amplification of ~400 bp of the V4-V5 hypervariable region of the 16S rRNA gene according to Earth Microbiome Project standards (EMP; http://www.earthmicrobiome.org/protocols-and-standards/16s/). Briefly, sample multiplexing was based on the EMP-proposed double-barcoding strategy using the EMP-recommended modifications (12-bp) Golay barcodes included on the forward primer 515F (5′-GTGYCAGCMGCCGCGGTAA) and additional 5-bp barcodes on the reverse primer 926R (5′-CCGYCAATTYMTTTRAGTTT) (69, 70). We also added a custom 18S rRNA gene blocking primer (named 926X [5′-GTGCCCTTCCGTCAATTCCT-C3 3′]) to counteract the low specificity of EMP primers toward the 16S rRNA gene 22. PCRs were carried out in triplicate, and successful amplification was confirmed with gel electrophoresis. Combined triplicates for each sample were purified with AMPure XP (Beckman Coulter) magnetic beads and equimolarly pooled into a single library (based on DNA concentration measured using a Synergy H1 [BioTek] spectrophotometer). The library was purified using Pippin Prep (Sage Science) from all fragments outside the 300- to 1,100-bp range. To control for contamination and PCR biases and to confirm barcoding success, we included four negative controls from the extraction procedure (ENCs), eight negative controls from the PCR process (NCs), and eight positive controls (PCs) of mock microbial communities. PCs were supplied commercially and comprised 4 samples of genomic DNA (gDNA) templates with equal abundance of 10 bacterial species (ATCC MSA-1000) and 4 samples with staggered abundance for the same bacteria (ATCC MSA-1001). Altogether, the library comprised PCR products from four 96-well plates, each containing one ENC, two NCs, and two different PCs. The library was sequenced in a single run of the Illumina MiSeq platform using v3 chemistry with 2 × 300-bp output (Norwegian High Throughput Sequencing Centre, Department of Medical Genetics, Oslo University Hospital).

### Data processing.

The sequencing process returned 15,893,914 reads. These raw reads were quality checked (FastQC [71]) and trimmed using USEARCH v9.2.64 (72) to keep the quality score above Q20. We trimmed the primers and demultiplexed and merged the reads, which resulted in a final amplicon length of 357 bp. We then clustered the reads at 100% identity for a representative set of sequences and used the USEARCH global alignment option at both 99% and 97% identity (72) for *de novo* OTU assignment. We subsequently used the BLAST algorithm (73) on the representative sequences, matching them against the SILVA 132 database (74) for taxonomic identification, producing a data set of 1,132 OTUs at 97% identity and 1,118 at 99% identity. We used the 97% identity OTU table as the primary data set and the 99% identity table as a supplemental data set to confirm that the patterns we found were not a product of identity threshold (see Fig. S1 in the supplemental material).

We recovered a mean of 16,898 reads per sample and a median of 14,751 reads (not including negative controls). From our positive controls, we recovered microbiome profiles that matched the expected community composition in each of the “staggered” and “even” mock communities. The two low-abundance species from the staggered templates (present at 0.04%) were successfully recovered from all four staggered mock samples. In the even mocks, there was a consistent overrepresentation of Clostridium beijerinckii and Escherichia coli (1.4× to 4.7× expected), leading to reductions in other taxa. Overall, the positive controls in this sequencing run matched our previous sequence outcomes (22, 75).

Any chloroplast, mitochondrial, or eukaryotic OTUs were identified in the OTU table and excluded. Potential bacterial contaminants were systematically evaluated using the R package decontam (v1.5.0) (76). The decontam package uses the prevalence or frequency of OTUs detected in negative controls to remove suspected contaminants. We used the prevalence method, which compares the presence or absence of each sequence in true-positive samples compared to the prevalence in negative controls. We used a strict threshold of 0.5, meaning any OTU with a higher proportion of reads in negative controls than test samples was excluded as a contaminant. The prevalence of each OTU is shown in Fig. S2. Forty-three OTUs were eliminated from the data set via this process, none of them normally recorded in *Drosophila* microbiomes. Singletons were also excluded, as was any OTU that made up less than 1% of the total reads for each sample, which collectively removed 837 OTUs. These procedures resulted in a data set of 110 OTUs and 360 samples.

### Statistical analyses.

Sample analysis was carried out using the packages vegan (77) and phyloseq (78) in R (79). To measure alpha diversity, we calculated the Shannon diversity index, using paired Wilcoxon and paired analysis of variance (ANOVA) tests to compare values between sets of samples. We calculated Bray-Curtis dissimilarity as a quantitative measure of beta diversity and used these values to create nonmetric multidimensional scaling ordinations (NMDS) to simultaneously evaluate the roles of elevation, site, *Drosophila* species, life stage, environment of origin, parasitism, trap identity, and duration of trap exposure (and isofemale line and generation number for laboratory samples). Where NMDS was deemed inappropriate by a mean stress value of >0.2, we used principal-coordinate analysis (PCoA) instead. To support these ordinations statistically, we calculated PERMANOVA tests on Bray-Curtis dissimilarity values. We applied the Benjamini-Hochberg correction on PERMANOVA *P* values to control for multiple comparisons and false discovery rate. We also evaluated the statistical significance of differences in dispersion among groups using multivariate homogeneity analysis (function betadisper in vegan) (77) and ran each of these tests with 999 permutations. In addition, we used DeSeq differential abundance analysis from the R package microbiomeSeq (80) to compare the relative abundances of bacterial taxa across different sample sets, e.g., differential abundance between *Drosophila* species or between sites.

To systematically order our analyses, we first tested every variable (environment of origin, elevation, gradient identity, species identity, developmental stage, trap identity, trap duration of exposure, generation number, isofemale line) with all samples to determine the relative importance of all studied factors (results shown below). We subsequently separated the data by environment of origin, i.e., we tested lab *Drosophila* samples separately and field *Drosophila* samples separately. Splitting the data set in this way meant some variables were not relevant to field samples and vice versa. For example, when analyzing field-only samples, we did not include isofemale line or generation number because these only pertained to lab samples. Similarly, when focusing on lab-only samples, we did not include trap identity, trap exposure time, or parasitoid status because none were parasitized, and traps were only a component of the field study. Furthermore, trap identity and site were correlated variables because each trap was only used at one site. We tested the food and bait samples separately because they were not focal samples and were used to support conclusions about *Drosophila* microbiomes. The only samples positive for parasitoid detection were from Drosophila rubida pupae; thus, we only compared these samples to other D. rubida pupae, and not as part of the core analyses.

### Data availability.

Raw sequence data are available on NCBI Sequence Read Archive (BioProject accession no. PRJNA849960).
